# Sequential cleavage and blastocyst embryo transfer and IVF outcomes: a systematic review

**DOI:** 10.1186/s12958-021-00824-y

**Published:** 2021-09-14

**Authors:** Jianeng Zhang, Chong Wang, Huanhuan Zhang, Yan Zhou

**Affiliations:** Reproductive Endocrinology Center, Hangzhou Women’s Hospital (Hangzhou Maternity and Child Health Care Hospital), Hangzhou, 310000 China

**Keywords:** Sequential embryo transfer, Cleavage embryo transfer, Blastocyst embryo transfer, *In vitro* fertilization, Systematic review

## Abstract

**Background:**

Sequential embryo transfer has been proposed as a way to improve embryo implantation in women for *in vitro* fertilization (IVF), but the effect on pregnancy outcomes remains ambiguous. This systematic review was conducted to investigate the efficacy of sequential embryo transfer on IVF outcomes.

**Methods:**

A literature search was performed in the PubMed, Web of Science, Cochrane Library, ScienceDirect and Wanfang databases. Data were pooled using a random- or fixed-effects model according to study heterogeneity. The results are expressed as relative risks (RRs) with 95% confidence intervals (CIs). Heterogeneity was evaluated by the I^2^ statistic. The study protocol was registered prospectively on INPLASY, ID: INPLASY202180019.

**Results:**

Ten eligible studies with 2658 participants compared sequential embryo transfer and cleavage transfer, while four studies with 513 participants compared sequential embryo transfer and blastocyst transfer. The synthesis results showed that the clinical pregnancy rate was higher in the sequential embryo transfer group than in the cleavage embryo transfer group (RR 1.42, 95% CI 1.26–1.60, *P*< 0.01) for both women who did experience repeated implantation failure (RIF) (RR 1.58, 95% CI 1.17–2.13, *P*< 0.01) and did not experience RIF (Non-RIF) (RR 1.44, 95% CI 1.20–1.66, *P*< 0.01). However, sequential embryo transfer showed no significant benefit over blastocyst embryo transfer.

**Conclusion:**

The current systematic review demonstrates that sequential cleavage and blastocyst embryo transfer improve the clinical pregnancy rate over conventional cleavage embryo transfer. For women with adequate embryos, sequential transfer could be attempted following careful consideration. More high-grade evidence from prospective randomized studies is warranted.

**Supplementary Information:**

The online version contains supplementary material available at 10.1186/s12958-021-00824-y.

## Background

*In vitro* fertilization and embryo transfer (IVF-ET) technology is an important choice for infertile couples. Ovulation induction protocols and embryo culture systems in the laboratory have been continuously optimized following decades of development, resulting in improved quantity and quality of embryos. However, the implantation rate remains 25–40%, preventing IVF-ET from having an ideal outcome [1].

Embryo implantation is a complex process involving multiple biological factors [2], requiring embryos with high developmental potential, good endometrial receptivity and effective dialogue between the two [3]. Determining how to adjust the embryo transfer strategy, make good use of existing embryos, and obtain ideal outcomes is a common problem faced by reproductive doctors and embryologists. In the early cleavage stage, the regulation of the embryonic genome is activated after Day 3 (8-cell stage). However, the developmental potential of current high-quality embryos, selected by morphology alone, cannot be accurately predicted [4]. Prolonging the culture time is a reliable method for naturally screening embryos with high developmental potential; therefore, blastocyst transplantation has a higher implantation rate and clinical pregnancy rate, but it increases the risk of cycle cancellation and reduces the chances of transplantation to a certain extent and is therefore not suitable for patients with fewer embryos [5, 6]. Currently, the transfer of embryos in two embryonic development stages in the same cycle, that is, two-step transfer with cleavage and blastocyst embryos in the same treatment cycle,is already performed in clinical practice. Sequential transfer [3] not only has a higher implantation rate than blastocysts transfer but also avoids the cancellation risk of the transfer cycle with previously transferred cleavage embryos [4]. However, there is no unified conclusion about the effect of sequential transplantation on IVF pregnancy outcomes.

This study sought to systematically review and summarize existing evidence related to the impact of sequential embryo transfer on IVF outcomes to further guide clinical transplantation strategies.

## Materials and methods

### Search strategy

In this systematic review, we searched the PubMed, Cochrane Library, Web of Science, ScienceDirect and Wanfang databases for studies published in the last two decades until January 2021 using a combination of MeSH terms and free words. The main search terms were ‘sequential embryo transfer’ or ‘consecutive embryo transfer’ or ‘sequential embryo transplantation’ or ‘sequencing embryo transfer’ or ‘interval double transfer’ or ‘two-step transfer’ and ‘IVF’ or ‘*in vitro* fertilization’. Language was restricted to English and Chinese in the searches.

### Eligible criteria and study selection

#### Inclusion criteria

We included randomized controlled trials, cohort studies and case-control studies that compared IVF outcomes between sequential transfer of cleavage- and blastocyst-stage embryosand regular embryo transfer on Day3 or Day5.

### Exclusion criteria

Studies published only as abstracts or repeated publications, as well as studies reportingon frozen-thawed embryo sequential transfer pregnancy outcomes, were excluded from this review.

### Study selection

**Fig. 1:**
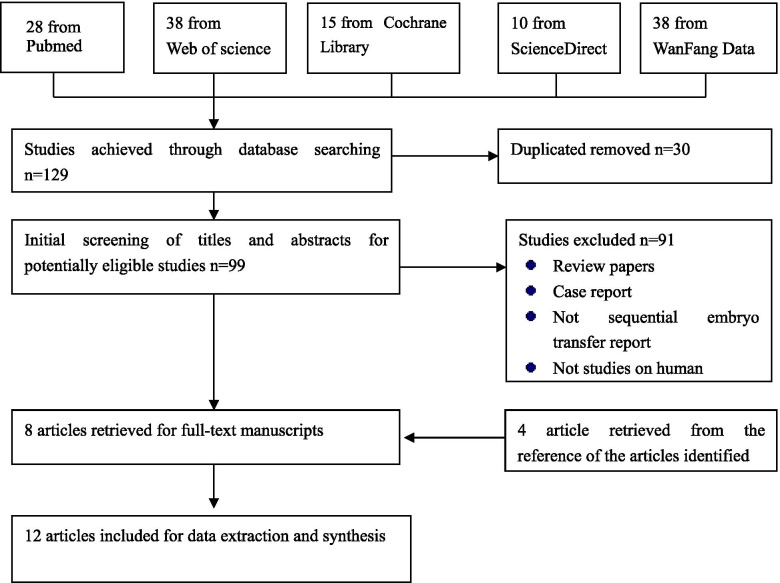
Study selection process for the systematic review

The titles and abstracts of the retrieved studies were screened independently by the two reviewers to identify studies for inclusion. Final inclusion or exclusion decisions and study quality assessments were made by examining the full manuscripts. A third reviewer was consulted to resolve any disagreement after discussion and consensus. The reference lists of the identified articles were screened for potential data resources. The study selection process for the systematic review is shown in Fig. 1.

### Study appraisal and data extraction

The methodological quality of all the selected studies was assessed. For randomized studies, information on the randomization method, allocation concealment, blinding, intention-to-treat analysis and follow-up rate was extracted [7] (Table S1). For cohort studies and case-control studies, the Newcastle–Ottawa Scale (NOS) was used for methodological quality appraisal [8] (Table S2).

For each study, data obtained from the manuscript included first author, year of publication, country of origin, study design, patient characteristics as age, investigations for repeated implantation failure (RIF), and embryo transfer protocol, etc.

### Statistical analysis

Study features and outcomes were assembled in a tabular form, and meta-analysis was performed using Review Manager 5.4.1 [9]. Study heterogeneity was tested by the chi-squared test and I^2^ test. *P* < 0.05 or I^2^> 40% was considered to indicate significant heterogeneity. Random-effects models were adopted when *P*< 0.05 or I^2^>40%; otherwise fixed-effects models were used. The effect estimate was expressed as the pooled RR with 95% CI. The outcome data were analysed separately according to the work-up of the participants (RIF/Non-RIF) if there was a difference among the population for one individual indicator between studies. Further sensitivity analysis was performed to assess the heterogeneity and outcome differences. Publication bias was assessed using funnel plots.

## Results

### Characteristics of included studies

A total of 129 articles were identified by the literature search. First, 30 duplicate articles were removed. After the initial screening of the titles and abstracts, 8 articles remained after excluding 91 unsuitable articles. In addition, 4 articles were retrieved from the references of the identified articles. Finally, 12 studies were included in this review and meta-analysis. Among them,8 articles reported the IVF outcomes of sequential embryo transfer versus cleavage embryo transfer; 2 reported the outcomes of sequential embryo transfer and blastocyst embryo transfer; and the last 2 compared the sequential transfer outcomes with those of both cleavage and blastocyst transfer contemporarily. As shown in Table 1, the study participants were from various parts of the world, including America, Africa, Asia, Europe and the Middle East. The pooled sample size was 2658 (1025 in observation group, 1633 in control group) in the comparison with cleavage transfer and513 (277 in observation group, 236 in control group) in the comparison with blastocyst transfer. The ages of the participants, RIF investigation and transfer protocols are also presented in Table 1.

**Table 1:** Characteristics of the 12 studies included in the meta-analysis

Author	Year	Country	Study design	RIF	Samples (n)		Age (mean±SD)		Embryo transfer protocol	
					Observation	Control	Observation	Control	Observation	Control
B.Almog [10]	2008	Israel	Retrospective Cohort Study	Y	65	66	34.3±0.7	34.7±0.1	D2/3 and D5 sequential embryo transfer	D2/3 embryo transfer
Simon J [11]	2003	Canada	Retrospective Cohort Study	N	110	35	34.7±3.5	32.0±4.4	Consecutive transfer	Single transfer
Wael A [12]	2015	Egypt	Randomized controlled trial	Y	74	73	34.4±1.4	34.0±1.5	Sequential D3/D5 embryo transfer	D3 embryo transfer
Cong Fang [13]	2012	China	Case Control Study	Y	66	D3:29 D5:85	34.1±3.2	D3:33.9±4.1 D5:33.1±4.5	Sequential D3/D5 embryo transfer	D3 embryo transfer/ D5 embryo transfer
KOICHI KYONO [14]	2003	Japan	Retrospective Cohort Study	Y	141	D3567 D5:105	35.4±4.5	D3:35.5±4.8 D5:35.±4.5	D3 and blastocyst sequential transfer	Early stage embryos/ blastocyst embryo transfer
RonitMachtinger [15]	2006	Israel	Retrospective Cohort Study	Y	66	117	30.7±3.2	31.0±2.9	Sequential transfer D3 and D 5/6 embryos	Transfer embryos on D3
Sakae Goto [16]	2005	Japan	Prospective Cohort Study	Y	90	90	37.4±5.1	37.7±4.8	Sequential transfer D2 and D5 embryos	D2embryos transfer
ChadiYazbeck [17]	2011	France	Retrospective Cohort Study	NS	120	280	33.1±4.1	33.6±3.9	D2/3 and D5/6 embryo sequential transfer	D2/3 embryo transfer
Gözde Kaya [18]	2020	Turkey	Prospective Cohort Study	N	53	135	29.0	31.0	D3 and D5 embryos sequential transfer	D2/3 embryo transfer
Jacob Ashkenazi [19]	2000	Israel	Retrospective Cohort Study	NS	136	139	31.1±4.9	32.2±5.6	Double transfer	Early transfer
EnsiehShahrokhTehraninejad [20]	2019	Iran	Randomized Clinical trial	Y	60	60	35.0 ± 4.4	34.1± 4.2	D3 and D5 embryos sequential transfer	D5 embryos transfer
D.Loutradis [21]	2004	Greece	Prospective Cohort Study	Y	44	42	34.95±4.9	36.2±0.7	2 embryo transfer on D2 and D4/5	Embryo transfer on D4/5

*Y* yes, *N* no, *NS* not suitable, *D3* day3, *D5* day5

### Quantitative data synthesis

#### Comparison between sequential transfer and cleavage embryo transfer

The statistical results between sequential transfer and cleavage embryo transfer have been listed in Table 2, while forest plots was shown in Figure S1.

**Table 2:** Summary of results of meta-analyses of comparison between sequential transfer and cleavage embryo transfer

Outcome indicator	Studies	Samples	Heterogeneity	Effect model	RR (95%CI)	*P* value
Chemical pregnancy	3	423	*P*=0.73 I^2^=0%	Fixed	1.59 (1.21-2.09)	<0.01*
Clinical pregnancy	10	2474	*P*=0.19 I^2^=28%	Fixed	1.42(1.26-1.60)	<0.01*
RIF	6	1440	*P*=0.03 I^2^=59%	Random	1.58 (1.17–2.13)	<0.01*
NRIF	4	1034	*P*=0.97 I^2^=0%	Random	1.44 (1.20–1.66)	<0.01*
Embryo implantation	4	3206	*P*<0.01 I^2^=89%	Random	1.67 (0.89–3.14)	0.11
Clinical miscarriage	5	517	*P*=0.19 I^2^=35%	Fixed	0.83(0.57-1.22)	0.35
RIF	3	315	*P*=0.39 I^2^=0%	Fixed	1.12 (0.71–1.77)	0.62
NRIF	2	202	*P*=0.34 I^2^=0%	Fixed	0.50 (0.25–1.02)	0.06
Multiple pregnancy	8	642	*P*=0.25 I^2^=23%	Fixed	1.10(0.83-1.47)	0.05
RIF	6	440	*P*=0.43 I^2^=0%	Fixed	1.47 (1.01–2.16)	0.05
NRIF	2	202	*P*=0.19 I^2^=35%	Fixed	0.72 ( 0.45–1.14)	0.16
Live birth	2	531	*P*=0.91 I^2^=0%	Fixed	1.99 (1.47–2.71)	<0.01*

*Abbreviations*: *NRIF* Non-RIF;*P<0.01

### Chemical pregnancy

Three studies (n=423) [10–12] reported serum human chorionic gonadotropin (HCG) levels. The results showed a statistically significant improvement in the chemical pregnancy rate in the sequential transfer group (RR=1.59, 95% CI 1.21–2.09, *P*< 0.01; Table 2).

### Clinical pregnancy

Ten studies (n=2474) [10–19] reported the clinical pregnancy rate. Six studies involving the RIF subgroup [10, 12–16] showed a statistically significant improvement in the clinical pregnancy rate in the sequential transfer group (RR 1.58, 95% CI 1.17–2.13, *P* < 0.01). Likewise, the four studies involving the non-RIF subgroup [11, 17–19] also showed a statistically significant improvement in the clinical pregnancy rate after sequential transfer (RR 1.44, 95% CI 1.20–1.66, *P* < 0.01; Table 2).

The heterogeneity test for subgroup differences showed that the χ^2^ value was 0.01, with df =1 and *P* = 0.93, while I^2^ was 0%, suggesting no statistical heterogeneity among the included studies between the RIF and non-RIF subgroups. The test for overall effect showed a statistically significant improvement in the clinical pregnancy rate after sequential transfer (Z=5.71, *P* < 0.01).

### Embryo implantation

Four of the eight studies [12–15], including 3206 participants, reported the embryo implantation rate. Pooling of the results from these four studies did not show a statistically significant improvement in embryo implantation after sequential transfer compared with cleavage embryo transfer (RR 1.67, 95% CI 0.89–3.14, *P* = 0.11; Table 2).

### Clinical miscarriage

Five studies (n=517) [11–14, 17] reported the clinical miscarriage rate or ongoing pregnancy rate. The three studies involving the RIF subgroup [12–14] showed no statistically significant difference in the clinical miscarriage rate between the sequential transfer group and the early embryo transfer group (RR 1.12, 95% CI 0.71–1.77, *P* = 0.62; Table 2). The remaining two studies, involving the Non-RIF subgroup [11, 17], showed a tendency of improvement in the clinical miscarriage rate after sequential transfer, but the difference was not statistically significant (RR 0.50, 95% CI 0.25–1.02, *P*< 0.06; Table 2). The heterogeneity test for subgroup differences showed that the χ^2^ value was 3.51, with df = 1 and *P* = 0.06, while I^2^ was 71.5%, suggesting high statistical heterogeneity between the RIF and non-RIF subgroups.

### Multiple pregnancy

Eight studies (n=642) [10–17] reported the multiple pregnancy rate. The six studies involving the RIF [10, 12–16] subgroup revealed a slight increase in the multiple pregnancy rate between the sequential and early embryo transfer groups, but the difference was not statistically significant (RR 1.47, 95% CI 1.01–2.16, *P* = 0.05). The two studies involving the non-RIF [11, 17] subgroup showed no significant difference between the sequential and early embryo transfer groups (RR 0.72, 95% CI 0.45–1.14, *P* = 0.16; Table 2). The heterogeneity test for subgroup differences showed that the χ^2^ value was 5.57, with df = 1 and *P* = 0.02, while I^2^ was 82.1%, suggesting high statistical heterogeneity between the RIF and non-RIF subgroups.

### Ectopic pregnancy

Only one study reported on ectopic pregnancy, which indicated that none occurred [17].

### Live birth

Two studies (n=531) [10, 17] reporting the live birth rate were included in the meta-analysis. The results showed a statistically significant improvement in the live birth rate forthe sequential transfer group (RR 1.99, 95% CI 1.47–2.71, *P*< 0.01; Table 2).

### Comparison between sequential transfer and blastocyst embryo transfer

The statistical results between sequential transfer and cleavage embryo transfer have been listed in Table 3, while forest plots was shown in Figure S2.

**Table 3:** Summary of results of meta-analyses of comparison between sequential transfer and blastocyst embryo transfer

Outcome indicator	Studies	Samples	Heterogeneity	Effect model	RR(95%CI)	*P* value
Clinical pregnancy	4	513	*P*=0.11I^2^=51%	Random	1.29 (0.90-1.85)	0.17
Embryo implantation	2	146	*P*=0.37I^2^=0%	Fixed	0.86 (0.64-1.17)	0.34
Clinical miscarriage	4	242	*P*=0.99I^2^=0%	Fixed	0.99 (0.68-1.44)	0.95
Multiple pregnancy	3	155	*P*=0.18I^2^=42%	Random	0.85 (0.47–1.52)	0.58

### Clinical pregnancy

Four studies (n=513) [13, 14, 20, 21] reporting the clinical pregnancy rate were included in the meta-analysis. The results showed no significant difference between the sequential transfer and blastocyst transfer groups under a random-effects model (RR 1.29, 95% CI 0.90–1.85, *P* = 0.17; Table 3).

### Embryo implantation

Two studies (n=807) [13, 14] that reported embryo implantation were included in the meta-analysis. The results showed no significant difference between the sequential transfer and blastocyst transfer groups under a fixed-effects model (RR 0.86, 95% CI 0.64–1.17, *P* = 0.34; Table 3).

### Clinical miscarriage

Four studies (n=242) [13, 14, 20, 21] comparing the clinical miscarriage rate between the sequential transfer and blastocyst transfer groups were included. The results showed no significant difference between the two groups under a fixed-effects model (RR 0.99, 95% CI 0.68–1.44, *P* = 0.95; Table 3).

### Multiple pregnancy

Three studies (n=155), [13, 14, 20] reporting the multiple pregnancy rate were included in the meta-analysis. Our results showed no significant difference between the sequential transfer and blastocyst transfer groups under a random-effects model (RR 0.85, 95% CI 0.47–1.52, *P* = 0.58; Table 3).

### Publication bias

**Fig. 2:**
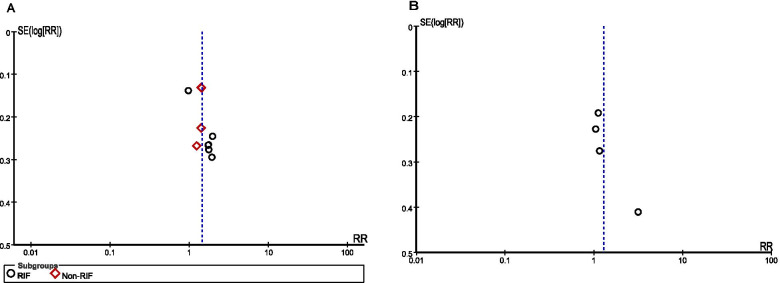
Funnel plot to assess publication and related biases in the systematic review. **A** Sequential transfer in comparison with cleavage embryo transfer; **B** Sequential transfer incomparison with blastocyst embryo transfer

Publication bias was assessed using funnel plots. The analysis results for publication and related biases did not suggest evidence of bias (Fig. 2).

### Sensitivity analysis

Generally, if the I^2^ test results exceed 40%, the heterogeneity is considered to be high. A random-effects model is used for analysis when I^2 ^exceeds 40%; otherwise, a fixed effects model is adopted. Sensitivity analysis was performed by sequentially excluding individual studies. Statistically similar results were obtained for each indication except after excluding KOICHI KYONO’s study while evaluating the sensitivity for the embryo implantation rate. Overall, I^2^ was 89%, but it declined to 6% (RR 2.17, 95% CI 1.64–2.89, *P*< 0.01) after deleting the study. This was likely due to the very large differences in patient selection and in the range of sample sizes. Therefore, data from this systematic analysis should be interpreted with caution until further high-grade evidence emerges.

## Discussion

Increasing the IVF success rate remains a clinical challenge. Different interventions have been proposed to improve pregnancy outcomes, but very few are directed towards embryo transfer. The present meta-analysis found that the positive impact of sequential transfer of embryos on the clinical pregnancy rate was consistent among the RIF and non-RIF subgroups with respect to conventional early embryo transfer, although the embryo implantation rate did not increase significantly. These results suggest that the increase in the clinical pregnancy rate in the sequential transfer group may be attributed to the second transfer of the blastocyst embryos. The lack of a significant increase in the clinical pregnancy rate or embryo implantation rate in the sequential transfer group relative to the blastocyst embryo transfer group also confirmed the hypothesis that blastocyst embryo transfer on Day 5 may account for a high proportion of the benefits to the pregnancy results. Blastocysts are well known to have high developmental potential, and the probability of transferring embryos with abnormal chromosomes decreases after prolonged culturing *in vitro*. Blastocyst transfer also increases the likelihood of synchronized endometrial and endometrial receptivity, thus increasing the implantation rate [4].

Alternatively, the findings may also indicate that early embryos have fewer chances to further grow after implantation in the endometrium than blastocyst embryos. However, in the IVF cycle, whether the culture should be extended remains controversial due to the risk of cancellation of the cycle in case a blastocyst is not obtained.

Some researchers have reported that two-thirds of IVF–embryo transfer failures are due to a lack of endometrial receptivity [22]. The endometrium becomes receptive to embryo implantation from 6 to 8 days after ovulation and remains receptive for 2 to 4 days. Different timings for the window of implantation have been confirmed based on transcriptomic modifications of the endometrium during the mid-luteal phase in at least 25% of RIF patients [23]. Researchers have even reported significantly higher embryo implantation and clinical pregnancy rates when simultaneously transferring Day 3 and Day 5 frozen embryos than when transferring two frozen blastocyst embryos in RIF patients [24]. Therefore, the variability in the endometrial maturation process and sequential transfer pinpointing the WOI, increasing the receptivity “window”, have been cited by the literature as the main factors for the increasing rates [10]. In addition, sequential transfer provides a mode for implanting good embryos cultured *in vivo* and synchronously *in vitro*, which may be one factor contributing to the higher clinical pregnancy rate [4].

In addition, sequential transfer itself involves two transfer procedures. The enhanced immune response triggered by the previously transferred cleavage embryo(s) stimulates an unknown adhesion factor [25–27], and the previously transferred embryo also cocultures with the endometrium, which is thought to create a better endometrial environment for the second transfer [4]. Additionally, a published meta-analysis revealed that the clinical pregnancy rate was significantly improved after local endometrial injury, suggesting that local mechanical microtrauma to the endometrium may activate structural and functional endometrial changes involving the stromal and epithelial components of the endometrium at the molecular level, consequently increasing endometrial receptivity [28]. Additionally, the first catheter insertion may cause similar stimulation to the endometrium [29].

The risk for multiple pregnancy is considerable due to the greater number of embryos transferred in the sequential embryo transfer procedure [30]. The multiple pregnancy rate from this review was not significantly different between the different types of transfer, in accordance with Wael’s study [12]. The increasing tendency of the multiple pregnancy rate in this review still indicates that the number of embryos sequentially transferred needs to be controlled rationally, taking age and the number of prior IVF failures into consideration. The clinical miscarriage rate did not differ significantly, which is not concordant with the concern that the second transfer procedure might have a deleterious influence [19], such as infection or trauma.

Embryo transfer is a key stage in IVF, in which the quality of the procedure determines the outcome. Ultrasound guidance of this procedure, which provides gynaecological imaging, is now also under debate. Transabdominal ultrasound guidance is currently used as the reference technique [31]. Recently, transvaginal ultrasound has emerged as an alternative for guiding embryo transfer, given its advances in imaging. Larue et al. reported that transvaginal ultrasound guidance of the transfer significantly increases the percentage of pregnancies per transfer, both in the general population and in the reference population, relative to transfers performed under transabdominal ultrasound guidance [32]. Markedly superior imaging resulting from the proximity of the target organs to the transducer, which thus leads to more precision and less trauma, and precision of embryo deposition may be the factors that account for the improvement in outcomes. Additionally, a systematic review concluded that transvaginal ultrasound and transabdominal ultrasoundwere comparable in terms of clinical pregnancy and ongoing or live birth rates. Nevertheless, the quality of evidence supporting the equivalence of the two approaches was low due to the small number of participants and some limitations in the study methodology [33]. The researcher suggested that if the two approaches were equivalent in terms of IVF outcomes, transvaginal ultrasound may be the first choice, as it is easier to perform (no second operator is needed), provides better visualization of the uterus andembryo transfer location, and leads to less patient pain, anxiety and discomfort [34].

The patient’s age and ovarian reserve are also highly related to the IVF outcome. To date, many efforts have been made to identify an algorithm that considers the patient’s age and ovarian reserve markers, such as anti-Müllerian hormone (AMH) and follicle-stimulating hormone, as a tool to optimize the recombinant follicle-stimulating hormone (rFSH) starting dose in the IVF procedure [35]. In addition, weight has also recently been taken into account, as research has revealed that to achieve equivalent clinical pregnancy rates, obese women require twice as much additional gonadotropins for ovarian stimulation as normal weight women [36]. However, not all the studies included in our review provided complete information in these respects. Although age was considered, the lack of ovarian reserve or weight evaluation led to inconclusive results. For those studies that did investigate ovarian reserve, comparisons between sequential transfer and cleavage transfer yielded consistent results, as two-step embryo transfer improved the clinical pregnancy rate [12, 13, 15, 16, 18]. However, when comparing sequential with blastocyst transfer, two studies demonstrated no significant difference [13, 20], while Loutradis et al [21] indicated that double embryo transfer had beneficial effects on patients with good embryos and previous failure attempts.

Overall, data from this systematic review should be interpreted with caution until further good-quality evidence from randomized trials emerges. The robustness of the results depends largely on the quality of the primary studies included in this review. First, the characteristics of the women recruited, such as age and ovarian reserve, were not homogenous among the included studies, leaving a number of pertinent questions unanswered. Correspondingly, ovarian response was not observed. Among the nonrandomized studies included, participants may have been distributed into a control group if they did not undergo blastocyst transfer, especially poor ovarian responders with insufficient embryos, which may have affected the study’s results. Second, we did not track unpublished articles or articles published more than two decades ago to obtain data for the analysis. The potential effect of this publication bias is unknown.

In conclusion, this meta-analysis demonstrates that sequential cleavage and blastocyst embryo transfer improves the clinical pregnancy rate over conventional cleavage embryo transfer. However, it showed no significant benefit over blastocyst embryo transfer. For women with adequate embryos, sequential transfer could be attempted following careful consideration. Overall, more high-grade evidence from prospective randomized studies is needed.

## Supplementary Information

**Additional file 1: Table S1**. Quality assessment by Newcastle–Ottawa Scale.

**Additional file 2: Table S2**. Quality assessment by Cochrane Handbook for Systematic Reviews of Interventions.

**Additional file 3: Figure S1**. Continued: Forest plots of comparison between sequential transfer and cleavage embryo transfer. Abbreviations: ST: sequential transfer; CT: cleavage transfer.

**Additional file 4: Figure S2**. Forest plots of comparison between sequential transfer and blastocyst transfer. Abbreviations: ST: sequential transfer; BT: blastocyst transfer.

## Data Availability

The current study was based on results of relevant published studies.

## Abbreviations

IVF: *in vitro* fertilization
RR: Relative risk
CI: Confidence interval
RIF: Repeated implantation failure
IVF-ET: *In vitro* fertilization and embryo transfer
NOS: Newcastle-Ottawa Scale
HCG: Human Chorionic Gonadotropin
AMH: Anti-Müllerian hormone
rFSH: Recombinant follicle-stimulating hormone
