# Supplementary material for: Sequential cleavage and blastocyst embryo transfer and IVF outcomes: a systematic review
Source: Reprod Biol Endocrinol. 2021 Sep 14;19:142. doi: 10.1186/s12958-021-00824-y (PMC8439041; doi:10.1186/s12958-021-00824-y)
Supplement: Supplementary file 1 — Additional file 1: Table S1. Quality assessment by Newcastle–Ottawa Scale. [file 12958_2021_824_MOESM1_ESM.docx]

| Included study | Selection | | | | Comparability | | Outcome | | | Total Stars |
| --- | --- | --- | --- | --- | --- | --- | --- | --- | --- | --- |
|  | Representativeness of the exposed cohort | Selection of the non exposed cohort | Ascertainment of exposure | Demonstration that outcome of interest was not present at start of study | Study controls for the most important factor | Study controls for second important factor | Assessment of outcome | Was follow-up long enough for outcomes to occur | Adequacy of follow up of cohorts |  |
| B.Almog 2008 | 1 | 1 | 1 | 1 | 1 | 1 | 1 | 1 | 1 | 9 |
| ChadiYazbeck 2011 | 1 | 1 | 1 | 1 | 1 | 0 | 1 | 1 | 1 | 8 |
| Simon J. 2003 | 1 | 1 | 1 | 1 | 1 | 0 | 1 | 1 | 1 | 8 |
| KOICHI KYONO 2003 | 1 | 1 | 1 | 1 | 1 | 0 | 1 | 1 | 1 | 8 |
| Jacob Ashkenazi 2000 | 1 | 1 | 1 | 1 | 1 | 1 | 1 | 1 | 1 | 9 |
| Sakae Goto 2005 | 1 | 1 | 1 | 1 | 1 | 0 | 1 | 1 | 1 | 8 |
| Cong Fang 2012 | 1 | 1 | 1 | 1 | 1 | 1 | 1 | 1 | 1 | 9 |
| RonitMachtinger 2006 | 1 | 1 | 1 | 1 | 1 | 1 | 1 | 1 | 1 | 9 |
| Loutradis D. 2004 | 1 | 1 | 1 | 1 | 1 | 1 | 1 | 1 | 1 | 9 |
| Gözde Kaya 2020 | 1 | 1 | 1 | 1 | 1 | 1 | 1 | 0 | 0 | 7 |

Table S1. Quality assessment by Newcastle–Ottawa Scale.
