# Supplementary material for: Sequential cleavage and blastocyst embryo transfer and IVF outcomes: a systematic review
Source: Reprod Biol Endocrinol. 2021 Sep 14;19:142. doi: 10.1186/s12958-021-00824-y (PMC8439041; doi:10.1186/s12958-021-00824-y)
Supplement: Supplementary file 2 — Additional file 2: Table S2. Quality assessment by Cochrane Handbook for Systematic Reviews of Interventions. [file 12958_2021_824_MOESM2_ESM.docx]

| Included study | Randomization method | Allocation concealment | Blinding | Intention to treat and follow up |
| --- | --- | --- | --- | --- |
| Wael A.  2015 | 2 | 2 | 0 | 1 |
| EnsiehShahrokh  Tehraninejad  2019 | 2 | 2 | 1 | 1 |

Table S2. Quality assessment by Cochrane Handbook for Systematic Reviews of Interventions.
