# Supplementary figures and images for: Sequential cleavage and blastocyst embryo transfer and IVF outcomes: a systematic review
Source: Reprod Biol Endocrinol. 2021 Sep 14;19:142. doi: 10.1186/s12958-021-00824-y (PMC8439041; doi:10.1186/s12958-021-00824-y)

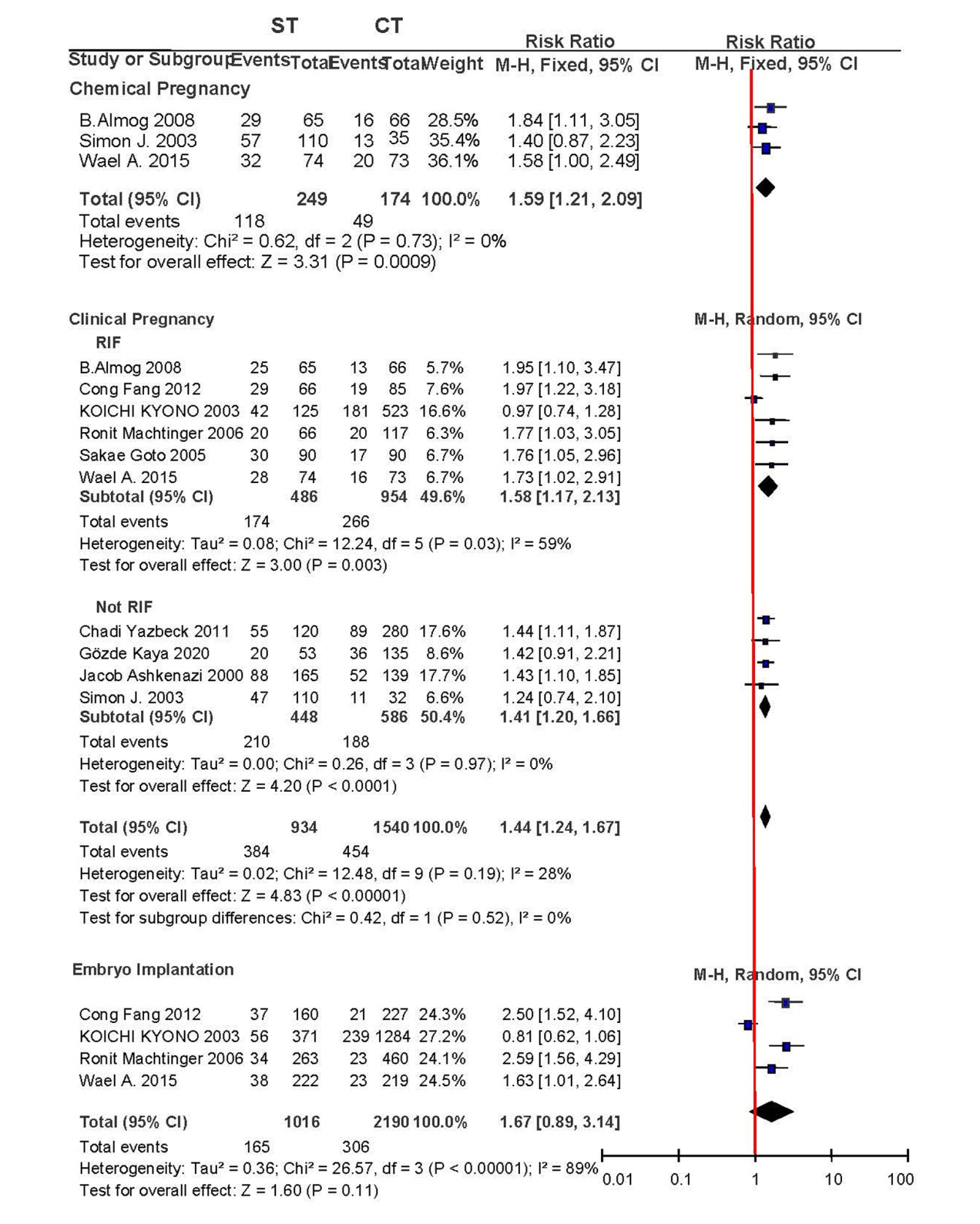


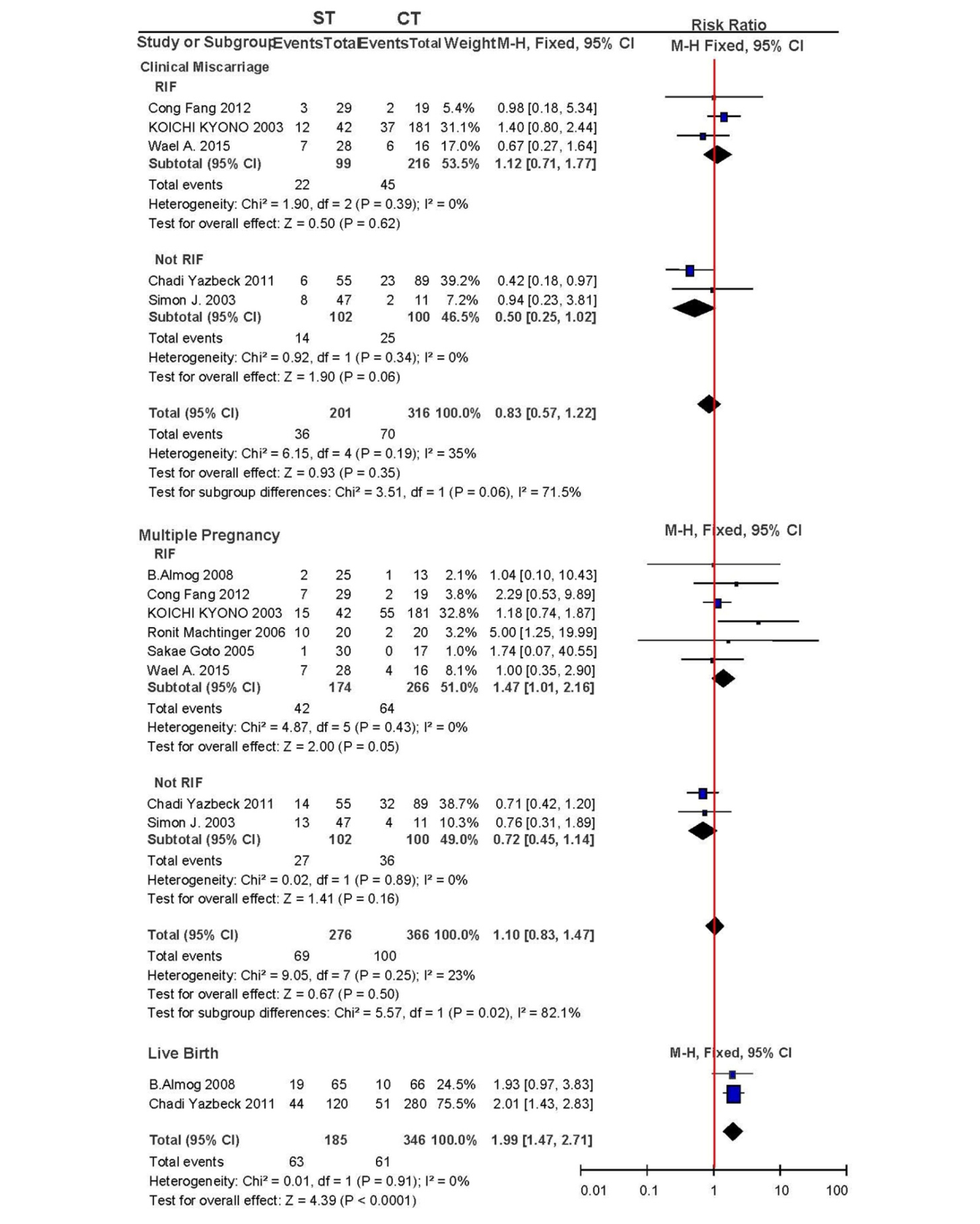

Supplement: Supplementary file 3 — Additional file 3: Figure S1. Continued: Forest plots of comparison between sequential transfer and cleavage embryo transfer. Abbreviations: ST: sequential transfer; CT: cleavage transfer. [file 12958_2021_824_MOESM3_ESM.docx]

**
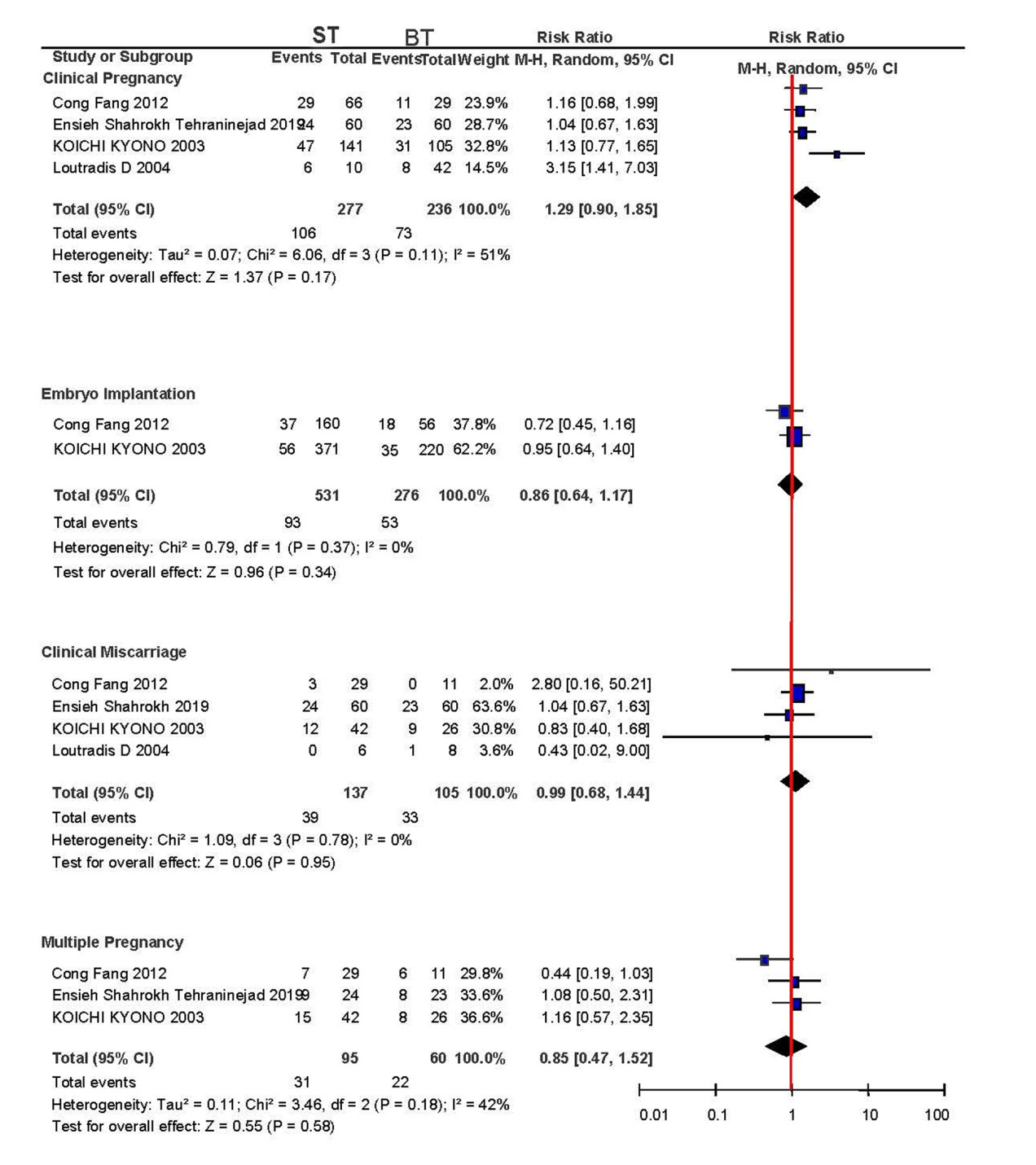
**

Supplement: Supplementary file 4 — Additional file 4: Figure S2. Forest plots of comparison between sequential transfer and blastocyst transfer. Abbreviations: ST: sequential transfer; BT: blastocyst transfer. [file 12958_2021_824_MOESM4_ESM.docx]
